# 2-Amino­anilinium benzoate

**DOI:** 10.1107/S1600536810048208

**Published:** 2010-11-24

**Authors:** Yan-wei Zhang

**Affiliations:** a Department of Chemical & Environmental Engineering, Anyang Institute of Technology, Anyang 455000, People’s Republic of China

## Abstract

In the crystal of the title molecular salt, C_6_H_9_N_2_^+^·C_7_H_5_O_2_^−^, the cations and anions are linked by N—H⋯O hydrogen bonds, buiding an *R*_2_^2^(9) ring. Futher N—H⋯O hydrogen bonds generate chains, which develop parallel to the *a* axis through the formation of *R*_4_^3^(10) rings.,

## Related literature

For the properties of amino compounds, see: Fu *et al.* (2009 ▶); Aminabhavi *et al.* (1986 ▶); Dai & Fu (2008**a* ▶,b* ▶). For hydrogen-bond motifs, see: Etter (1990 ▶); Bernstein *et al.* (1995 ▶).
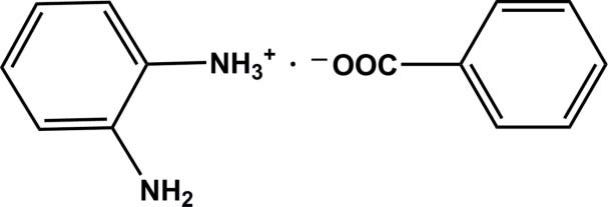


## Experimental

### 

#### Crystal data

C_6_H_9_N_2_^+^·C_7_H_5_O_2_^−^
*M* *_r_* = 230.26
Orthorhombic, 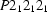

*a* = 6.0211 (12) Å
*b* = 12.237 (2) Å
*c* = 16.639 (3) Å
*V* = 1226.0 (4) Å^3^
*Z* = 4
Mo *K*α radiation
μ = 0.09 mm^−1^
*T* = 298 K
0.30 × 0.15 × 0.10 mm

#### Data collection

Rigaku Mercury2 diffractometer
Absorption correction: multi-scan (*CrystalClear*; Rigaku, 2005 ▶) *T*_min_ = 0.970, *T*_max_ = 1.000
12656 measured reflections
1638 independent reflections
1133 reflections with *I* > 2σ(*I*)
*R*_int_ = 0.053

#### Refinement

*R*[*F* ^2^ > 2σ(*F* ^2^)] = 0.050
*wR*(*F* ^2^) = 0.118
*S* = 1.08
1638 reflections
155 parameters
H-atom parameters constrained
Δρ_max_ = 0.11 e Å^−3^
Δρ_min_ = −0.16 e Å^−3^

### 

Data collection: *CrystalClear* (Rigaku, 2005 ▶); cell refinement: *CrystalClear*; data reduction: *CrystalClear*; program(s) used to solve structure: *SHELXS97* (Sheldrick, 2008 ▶); program(s) used to refine structure: *SHELXL97* (Sheldrick, 2008 ▶); molecular graphics: *ORTEPIII* (Burnett & Johnson, 1996 ▶), *ORTEP-3 for Windows* (Farrugia, 1997 ▶) and *PLATON* (Spek, 2009 ▶); software used to prepare material for publication: *SHELXL97*.

## Supplementary Material

Crystal structure: contains datablocks I, global. DOI: 10.1107/S1600536810048208/dn2626sup1.cif

Structure factors: contains datablocks I. DOI: 10.1107/S1600536810048208/dn2626Isup2.hkl

Additional supplementary materials:  crystallographic information; 3D view; checkCIF report

## Figures and Tables

**Table 1:** Hydrogen-bond geometry (Å, °)

*D*—H⋯*A*	*D*—H	H⋯*A*	*D*⋯*A*	*D*—H⋯*A*
N1—H1*C*⋯O1	0.89	1.83	2.699 (3)	166
N2—H2*A*⋯O2	1.00	2.16	3.093 (3)	155
N1—H1*B*⋯O1^i^	0.89	1.94	2.815 (3)	168
N1—H1*A*⋯O2^ii^	0.89	1.90	2.753 (3)	161

Symmetry codes: (i) 
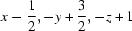
; (ii) 
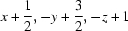
.

**Fig. 1.**
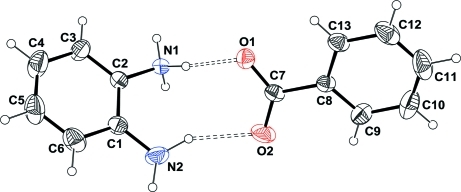
A view of the title compound with the atomic numbering scheme. Displacement ellipsoids are drawn at the 30% probability level. H atoms are represented as small spheres of arbitrary radii. H bonds are shown as dashed lines.

**Fig. 2.**
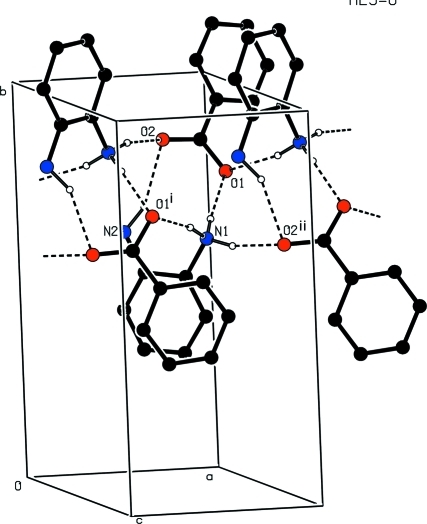
Partial packing view of the title compound showing the formation of the chain through the R34(10) rings. H atoms not involved in hydrogen bonding have been omitted for clarity. H bonds are shown as dashed lines. [Symmetry codes: (i) -x-1, y+3/2, -z+3/2; (ii) -x, y+3/2, -z+3/2]

## supplementary crystallographic information

### Comment

The amino derivatives have found wide range of applications in material science, such as magnetic, fluorescent and dielectric behaviors. And there has been an increased interest in the preparation of amino coordination compound (Aminabhavi *et al.*, 1986; Dai & Fu 2008*a*; Dai & Fu 2008*b*; Fu, *et al.* 2009). As an extension on the structural characterization, we report here the crystal structure of the title compound 2-aminoanilinium benzoate.

The asymmetrical unit of the title compound consists of one benzene-1,2-diamine cation and one benzoic acid anion linked by N-H···O hydrogen bonds buiding a R_2_^2^(9) ring (Etter, 1990, Bernstein *et al.*, 1995) (Fig.1; Table 1). As expected The carboxyl acid group has protonated one of the amine N atom.

N—H···O hydrogen bonds generate chains which develop parallel to the a axis through the formation of R_3_^4^(10) ring (Etter, 1990; Bernstein *et al.*, 1995)(Fig. 2; Table 1).

### Experimental

A mixture of benzene-1,2-diamine (0.1 mmol) and benzoic acid (0.1 mmol) was dissolved in ethanol (20 ml). The solution was allowed to evaporate to obtain colourless block-shaped crystals of the title compound.

### Refinement

All H atoms attached to C atoms and N(NH_3_) were fixed geometrically and treated as riding with C—H = 0.93 Å and N-H = 0.85Å with *U*_iso_(H) = 1.2*U*_eq_(C) or *U*_iso_(H) = 1.5*U*_eq_(N) . H atoms bonded to N(NH_2_) atom were located in difference Fourier maps and their coordinates were refined using restraints (N-H=1.00 (1)Å; H···H = 1.80 (2) Å)  with *U*_iso_(H) = 1.5*U*_eq_(N). In the last cycle of refinement they were treated as riding on the N.

In the absence of significant anomalous scattering, the absolute configuration could not be reliably determined and then the Friedel pairs were merged and any references to the Flack parameter were removed.

### Crystal data

**Table:**

C_6_H_9_N_2_^+^·C_7_H_5_O_2_^−^	*F*(000) = 488
*M**_r_* = 230.26	*D*_x_ = 1.247 Mg m^−^^3^
Orthorhombic, *P*2_1_2_1_2_1_	Mo *K*α radiation, λ = 0.71073 Å
Hall symbol: P 2ac 2ab	Cell parameters from 2805 reflections
*a* = 6.0211 (12) Å	θ = 3.3–27.5°
*b* = 12.237 (2) Å	µ = 0.09 mm^−^^1^
*c* = 16.639 (3) Å	*T* = 298 K
*V* = 1226.0 (4) Å^3^	Block, colourless
*Z* = 4	0.30 × 0.15 × 0.10 mm

### Data collection

**Table:**

Rigaku Mercury2 diffractometer	1638 independent reflections
Radiation source: fine-focus sealed tube	1133 reflections with *I* > 2σ(*I*)
graphite	*R*_int_ = 0.053
Detector resolution: 13.6612 pixels mm^-1^	θ_max_ = 27.5°, θ_min_ = 3.3°
CCD profile fitting scans	*h* = −7→7
Absorption correction: multi-scan (*CrystalClear*; Rigaku, 2005)	*k* = −15→15
*T*_min_ = 0.970, *T*_max_ = 1.000	*l* = −21→21
12656 measured reflections	

### Refinement

**Table:**

Refinement on *F*^2^	Primary atom site location: structure-invariant direct methods
Least-squares matrix: full	Secondary atom site location: difference Fourier map
*R*[*F*^2^ > 2σ(*F*^2^)] = 0.050	Hydrogen site location: inferred from neighbouring sites
*wR*(*F*^2^) = 0.118	H-atom parameters constrained
*S* = 1.08	*w* = 1/[σ^2^(*F*_o_^2^) + (0.0536*P*)^2^ + 0.0477*P*] where *P* = (*F*_o_^2^ + 2*F*_c_^2^)/3
1638 reflections	(Δ/σ)_max_ = 0.002
155 parameters	Δρ_max_ = 0.11 e Å^−^^3^
0 restraints	Δρ_min_ = −0.16 e Å^−^^3^

### Special details

**Table:**

Geometry. All esds (except the esd in the dihedral angle between two l.s. planes) are estimated using the full covariance matrix. The cell esds are taken into account individually in the estimation of esds in distances, angles and torsion angles; correlations between esds in cell parameters are only used when they are defined by crystal symmetry. An approximate (isotropic) treatment of cell esds is used for estimating esds involving l.s. planes.
Refinement. Refinement of *F*^2^ against ALL reflections. The weighted *R*-factor *wR* and goodness of fit *S* are based on *F*^2^, conventional *R*-factors *R* are based on *F*, with *F* set to zero for negative *F*^2^. The threshold expression of *F*^2^ > σ(*F*^2^) is used only for calculating *R*-factors(gt) *etc*. and is not relevant to the choice of reflections for refinement. *R*-factors based on *F*^2^ are statistically about twice as large as those based on *F*, and *R*- factors based on ALL data will be even larger.

### Fractional atomic coordinates and isotropic or equivalent isotropic displacement parameters (Å^2^)

**Table:**

	*x*	*y*	*z*	*U*_iso_*/*U*_eq_	
N1	0.7176 (3)	0.63438 (15)	0.49671 (11)	0.0429 (5)	
H1A	0.8339	0.6156	0.5267	0.064*	
H1B	0.6171	0.6679	0.5272	0.064*	
H1C	0.7617	0.6793	0.4578	0.064*	
N2	0.3415 (4)	0.65071 (19)	0.39767 (14)	0.0658 (7)	
H2A	0.4535	0.7109	0.3927	0.099*	
H2B	0.2137	0.6471	0.3597	0.099*	
C1	0.4321 (4)	0.5481 (2)	0.41353 (14)	0.0475 (6)	
C2	0.6195 (4)	0.53644 (19)	0.46122 (13)	0.0417 (6)	
C3	0.7065 (5)	0.4356 (2)	0.47948 (17)	0.0601 (8)	
H3	0.8326	0.4301	0.5115	0.072*	
C4	0.6058 (8)	0.3428 (2)	0.4501 (2)	0.0846 (11)	
H4	0.6638	0.2743	0.4619	0.101*	
C5	0.4195 (8)	0.3522 (3)	0.4032 (2)	0.0845 (10)	
H5	0.3511	0.2895	0.3836	0.101*	
C6	0.3325 (6)	0.4528 (3)	0.38492 (18)	0.0706 (9)	
H6	0.2059	0.4576	0.3531	0.085*	
O1	0.8819 (3)	0.79093 (13)	0.39910 (9)	0.0512 (5)	
O2	0.5619 (3)	0.87900 (18)	0.39486 (13)	0.0749 (7)	
C7	0.7644 (4)	0.8731 (2)	0.38177 (14)	0.0450 (6)	
C8	0.8786 (4)	0.9670 (2)	0.34141 (13)	0.0416 (6)	
C9	0.7821 (6)	1.0692 (2)	0.33900 (17)	0.0630 (8)	
H9	0.6427	1.0799	0.3618	0.076*	
C10	0.8903 (8)	1.1555 (3)	0.3032 (2)	0.0858 (11)	
H10	0.8253	1.2244	0.3029	0.103*	
C11	1.0919 (8)	1.1402 (3)	0.2682 (2)	0.0893 (12)	
H11	1.1642	1.1986	0.2438	0.107*	
C12	1.1892 (5)	1.0385 (3)	0.26864 (17)	0.0764 (10)	
H12	1.3254	1.0278	0.2435	0.092*	
C13	1.0847 (4)	0.9529 (2)	0.30625 (14)	0.0535 (7)	
H13	1.1531	0.8848	0.3081	0.064*	

### Atomic displacement parameters (Å^2^)

**Table:**

	*U*^11^	*U*^22^	*U*^33^	*U*^12^	*U*^13^	*U*^23^
N1	0.0360 (10)	0.0443 (11)	0.0485 (11)	0.0012 (10)	−0.0044 (9)	0.0033 (9)
N2	0.0584 (14)	0.0684 (15)	0.0706 (15)	0.0104 (13)	−0.0274 (14)	−0.0004 (12)
C1	0.0447 (14)	0.0537 (16)	0.0441 (13)	−0.0013 (14)	−0.0019 (12)	−0.0034 (12)
C2	0.0403 (13)	0.0442 (13)	0.0405 (12)	−0.0028 (13)	0.0009 (12)	−0.0013 (11)
C3	0.070 (2)	0.0463 (15)	0.0638 (17)	0.0067 (16)	−0.0119 (16)	0.0006 (14)
C4	0.117 (3)	0.0464 (17)	0.090 (2)	0.004 (2)	−0.018 (3)	−0.0056 (16)
C5	0.117 (3)	0.057 (2)	0.080 (2)	−0.022 (2)	−0.015 (2)	−0.0091 (17)
C6	0.073 (2)	0.077 (2)	0.0612 (17)	−0.0195 (18)	−0.0146 (17)	−0.0081 (16)
O1	0.0547 (11)	0.0483 (10)	0.0506 (10)	−0.0027 (9)	0.0006 (10)	0.0044 (8)
O2	0.0383 (10)	0.1060 (17)	0.0804 (14)	−0.0014 (11)	0.0130 (11)	0.0259 (13)
C7	0.0371 (13)	0.0587 (16)	0.0393 (13)	−0.0023 (14)	0.0003 (11)	0.0004 (13)
C8	0.0395 (13)	0.0518 (14)	0.0334 (11)	0.0025 (13)	−0.0023 (11)	0.0032 (11)
C9	0.0675 (19)	0.0622 (18)	0.0592 (16)	0.0089 (17)	−0.0015 (16)	0.0070 (15)
C10	0.125 (3)	0.0573 (18)	0.075 (2)	−0.001 (2)	−0.013 (3)	0.0192 (17)
C11	0.109 (3)	0.095 (3)	0.063 (2)	−0.046 (3)	−0.016 (2)	0.032 (2)
C12	0.058 (2)	0.117 (3)	0.0539 (17)	−0.022 (2)	0.0012 (15)	0.022 (2)
C13	0.0426 (14)	0.0721 (18)	0.0458 (14)	−0.0039 (15)	0.0010 (13)	0.0107 (13)

### Geometric parameters (Å, °)

**Table:**

N1—C2	1.461 (3)	C6—H6	0.9300
N1—H1A	0.8900	O1—C7	1.263 (3)
N1—H1B	0.8900	O2—C7	1.241 (3)
N1—H1C	0.8900	C7—C8	1.499 (3)
N2—C1	1.394 (3)	C8—C9	1.379 (4)
N2—H2A	1.0021	C8—C13	1.383 (4)
N2—H2B	0.9968	C9—C10	1.376 (5)
C1—C2	1.387 (4)	C9—H9	0.9300
C1—C6	1.395 (4)	C10—C11	1.359 (6)
C2—C3	1.375 (3)	C10—H10	0.9300
C3—C4	1.377 (4)	C11—C12	1.375 (5)
C3—H3	0.9300	C11—H11	0.9300
C4—C5	1.371 (6)	C12—C13	1.373 (4)
C4—H4	0.9300	C12—H12	0.9300
C5—C6	1.373 (5)	C13—H13	0.9300
C5—H5	0.9300		
			
C2—N1—H1A	109.5	C5—C6—C1	120.7 (3)
C2—N1—H1B	109.5	C5—C6—H6	119.7
H1A—N1—H1B	109.5	C1—C6—H6	119.7
C2—N1—H1C	109.5	O2—C7—O1	123.9 (3)
H1A—N1—H1C	109.5	O2—C7—C8	119.0 (3)
H1B—N1—H1C	109.5	O1—C7—C8	117.1 (2)
C1—N2—H2A	114.5	C9—C8—C13	118.7 (3)
C1—N2—H2B	112.5	C9—C8—C7	121.0 (2)
H2A—N2—H2B	120.0	C13—C8—C7	120.3 (2)
C2—C1—N2	121.3 (2)	C10—C9—C8	120.6 (3)
C2—C1—C6	117.3 (2)	C10—C9—H9	119.7
N2—C1—C6	121.4 (2)	C8—C9—H9	119.7
C3—C2—C1	121.9 (2)	C11—C10—C9	120.2 (3)
C3—C2—N1	119.6 (2)	C11—C10—H10	119.9
C1—C2—N1	118.4 (2)	C9—C10—H10	119.9
C2—C3—C4	119.6 (3)	C10—C11—C12	120.2 (3)
C2—C3—H3	120.2	C10—C11—H11	119.9
C4—C3—H3	120.2	C12—C11—H11	119.9
C5—C4—C3	119.6 (3)	C13—C12—C11	119.9 (3)
C5—C4—H4	120.2	C13—C12—H12	120.1
C3—C4—H4	120.2	C11—C12—H12	120.1
C4—C5—C6	120.9 (3)	C12—C13—C8	120.5 (3)
C4—C5—H5	119.6	C12—C13—H13	119.7
C6—C5—H5	119.6	C8—C13—H13	119.7
			
N2—C1—C2—C3	177.9 (2)	O1—C7—C8—C9	−163.0 (2)
C6—C1—C2—C3	0.5 (4)	O2—C7—C8—C13	−162.2 (2)
N2—C1—C2—N1	1.7 (4)	O1—C7—C8—C13	16.5 (3)
C6—C1—C2—N1	−175.7 (2)	C13—C8—C9—C10	−0.8 (4)
C1—C2—C3—C4	−0.2 (4)	C7—C8—C9—C10	178.7 (3)
N1—C2—C3—C4	176.0 (3)	C8—C9—C10—C11	1.5 (5)
C2—C3—C4—C5	−0.3 (5)	C9—C10—C11—C12	−0.3 (5)
C3—C4—C5—C6	0.3 (6)	C10—C11—C12—C13	−1.5 (5)
C4—C5—C6—C1	0.1 (5)	C11—C12—C13—C8	2.2 (4)
C2—C1—C6—C5	−0.5 (4)	C9—C8—C13—C12	−1.0 (4)
N2—C1—C6—C5	−177.8 (3)	C7—C8—C13—C12	179.5 (2)
O2—C7—C8—C9	18.3 (4)		

### Hydrogen-bond geometry (Å, °)

**Table:**

*D*—H···*A*	*D*—H	H···*A*	*D*···*A*	*D*—H···*A*
N1—H1C···O1	0.89	1.83	2.699 (3)	166
N2—H2A···O2	1.00	2.16	3.093 (3)	155
N1—H1B···O1^i^	0.89	1.94	2.815 (3)	168
N1—H1A···O2^ii^	0.89	1.90	2.753 (3)	161

Symmetry codes: (i) *x*−1/2, −*y*+3/2, −*z*+1; (ii) *x*+1/2, −*y*+3/2, −*z*+1.
